# Application of text-mining for updating protein post-translational modification annotation in UniProtKB

**DOI:** 10.1186/1471-2105-14-104

**Published:** 2013-03-22

**Authors:** Anne-Lise Veuthey, Alan Bridge, Julien Gobeill, Patrick Ruch, Johanna R McEntyre, Lydie Bougueleret, Ioannis Xenarios

**Affiliations:** 1 Swiss-Prot group, SIB Swiss Institute of Bioinformatics, 1 Michel Servet, Geneva 4, 1211, Switzerland; 2 BiTeM Group, Information Science Department, University of Applied Sciences, Carouge, 1227, Switzerland; 3 EMBL-European Bioinformatics Institute, Wellcome Trust Genome Campus, Hinxton, Cambridge, CB10 1SD, UK; 4 Vital-IT group, SIB Swiss Institute of Bioinformatics, Quartier Sorge, Bâtiment Génopode, Lausanne, 1015, Switzerland; 5 University of Lausanne, Lausanne, 1015, Switzerland

## Abstract

**Background:**

The annotation of protein post-translational modifications (PTMs) is an important task of UniProtKB curators and, with continuing improvements in experimental methodology, an ever greater number of articles are being published on this topic. To help curators cope with this growing body of information we have developed a system which extracts information from the scientific literature for the most frequently annotated PTMs in UniProtKB.

**Results:**

The procedure uses a pattern-matching and rule-based approach to extract sentences with information on the type and site of modification. A ranked list of protein candidates for the modification is also provided. For PTM extraction, precision varies from 57% to 94%, and recall from 75% to 95%, according to the type of modification. The procedure was used to track new publications on PTMs and to recover potential supporting evidence for phosphorylation sites annotated based on the results of large scale proteomics experiments.

**Conclusions:**

The information retrieval and extraction method we have developed in this study forms the basis of a simple tool for the manual curation of protein post-translational modifications in UniProtKB/Swiss-Prot. Our work demonstrates that even simple text-mining tools can be effectively adapted for database curation tasks, providing that a thorough understanding of the working process and requirements are first obtained. This system can be accessed at http://eagl.unige.ch/PTM/.

## Background

Post-translational modifications (PTMs) of protein sequences regulate many aspects of protein behavior including protein activity, localization, interactions, and expression level, and their characterization is a crucial step towards the complete description of protein function. Databases such as the UniProt KnowledgeBase (UniProtKB) [1] include information on PTMs that is directly curated from the scientific literature. The continuing and ever-accelerating growth of this literature presents a challenge for database curators (and scientists) who wish to keep pace with the knowledge on PTMs and their functions. Text mining tools may facilitate the work of database curators in three ways, through: (1) the identification and prioritization of relevant documents, (2) the identification of bio-entity mentions in the text of those documents, such as gene and disease names, as well as their normalization (meaning the linking of these entities to database identifiers or ontology terms), and (3) the extraction of biological events, which necessitates determination of the relationships between entities [2]. Biological event extraction has been the subject of increasing interest by the text mining community in recent years, with typical challenges relating to the extraction of information on protein-protein interactions or PTMs. Examples include the community-based evaluations of event extraction methods in the BioCreative II/III challenges (for protein-protein interaction extraction) [3], and the BioNLP Shared Tasks, which included PTM event extraction for phosphorylation (BioNLP 2009) [4], and 5 additional PTMs (BioNLP 2011) [5]. The results of these BioNLP challenges were very promising, with participants achieving F-scores over 80% for single PTM type extraction [6] and approaching 70% for multiple PTMs [7]. The highest-performing methods were based on natural language processing (NLP) and machine-learning techniques. In the follow-up to the BioNLP Shared Tasks, S Pyysalo et al. [8] extended the event extraction task to 40 PTMs, but system performance decreased with the best systems achieving F-scores only slightly in excess of 50%. PTM information extraction methods were also developed in other contexts. RLIMS-P was the first tool for protein phosphorylation information extraction [9,10]. This tool processes documents with a shallow parser and then extracts information by matching text with manually designed patterns. eFIP is a system produced by the developers of RLIMS-P which combines various tools to identify abstracts which provide information on the functional context of protein phosphorylation, such as protein interaction induced by phosphorylation [11]. Another tool, MinePhos, uses enhanced RLIMS-P patterns along with support vector machines (SVM) combined with dictionary lookup to identify the modified protein [12]. These methods achieved good performance with precision and recall over 80%, but they were not assessed on the same dataset as the tools participating in the BioNLP challenges.

In this work, we developed a PTM information extraction procedure to assist curation of UniProtKB. UniProtKB is a high-quality resource of protein sequence and functional information that includes substantial curated information on protein sequence features, including PTMs [13]. To support UniProt curation efforts we developed an in-house system that is able to extract the information that is needed for UniProtKB PTM annotation from PubMed abstracts. The tool was developed in close collaboration with UniProtKB curators. Since PTM data are often not displayed in abstracts, we also explored the extraction of PTM information from full-text articles. The tool is based on a pattern-matching and rule-based approach, and specifically targets those PTMs that are most frequently annotated in UniProtKB, namely acetylation, amidation, disulfide bridge formation, glycosylation, methylation, phosphorylation, and sulfation. Our procedure is used to continually track new articles describing modified sites as they appear in PubMed. It is also used in a targeted fashion, to find potential confirmatory evidence for sites that have been annotated in UniProtKB based on information from high-throughput mass spectrometry-based proteomic studies [14]. Since such studies do not normally include significant follow-up or functional studies on potential identified sites, we use our tool to scan the literature to find new studies which may provide these types of supporting information.

## Results

**Figure 1:**
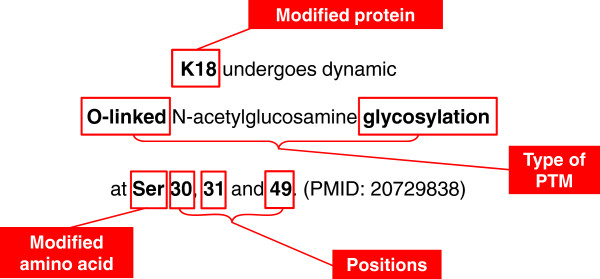
**A typical sentence with information on protein glycosylation:** Boxes indicate the information that is extracted from the sentence.

**Figure 2:**
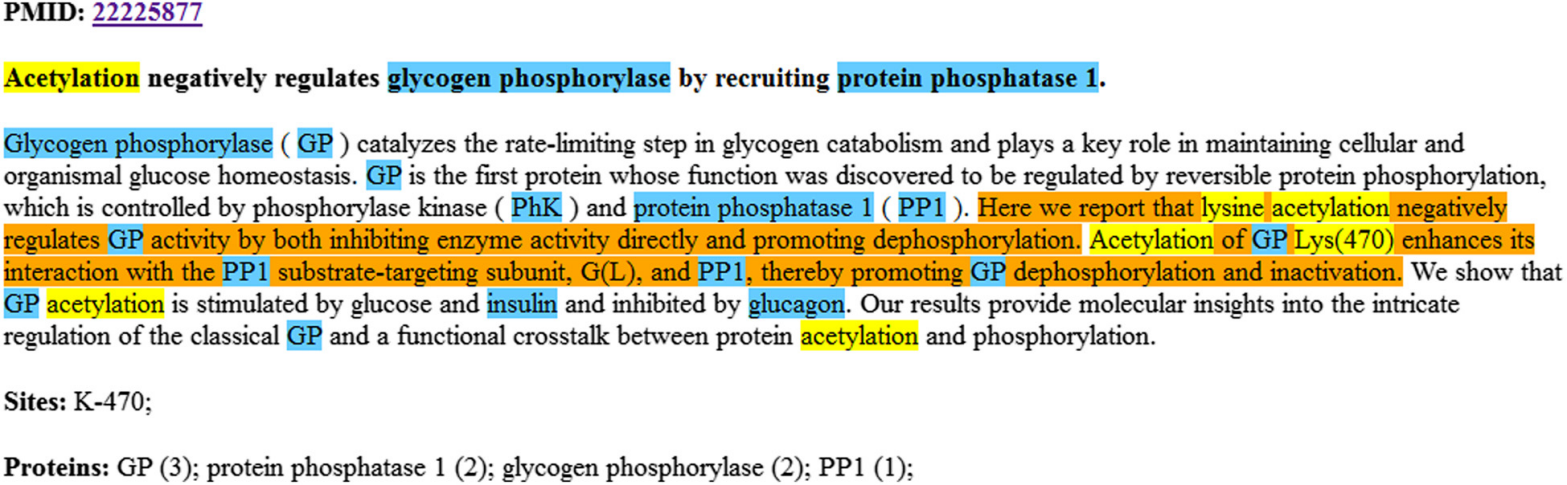
**An abstract containing information relevant to protein acetylation:** the extracted sentences are highlighted in orange, PTM and site information in yellow, and gene/protein mentions in blue. The list of extracted sites and proteins with scores are also provided. The two last sentences which mention acetylation are not highlighted since they contain no site information.

The system we developed is designed to facilitate the annotation of UniProtKB with information on post-translational modifications (PTMs). Such annotation requires knowledge of (1) the type of modification, (2) the modified amino acid and its position in the protein sequence, and (3) the protein which is modified (Figure 1). In UniProtKB, over 300 different PTMs are described. Most of these are relatively rare, so for development purposes we limited the scope of the tool to the most frequently annotated PTMs, namely acetylation, amidation, disulfide bond formation, glycosylation, methylation, phosphorylation, and sulfation. Using a pattern-matching and rule-based approach, the procedure retrieves information in a stepwise fashion, retaining those sentences which contain both information on the actual type of PTM and the identity of the modified site. The type of PTM is detected in two steps. First, the whole document (either abstract or article’s section when full-text is processed) is screened for the presence of generic tokens specific for each PTM (listed in Table 1, “Filtering token”). Following this initial triage, each sentence of the document is checked with a set of fine-grained regular expressions related to the generic token that matches (see Additional file 1 for details). In the retained sentences, the modification site is sought for the mention of at least an amino acid which can be modified. This is achieved using regular expressions and does not require any syntactic link between terms describing site and PTM type; hence we avoid the use of deep NLP-parsing and the design of complex extraction pattern templates specific for each PTM. For the detection of the position in the sequence, however, we used preposition-based parsing [15,16] which relies on finding specific grammatical elements, like preposition, punctuation or conjunction, which link amino acid mentions and figures. For instance, in the sentence “LC-MS/MS analysis of PKCdelta-activated intact hBVR identified phosphorylated serine positions 21, 33, 230, and 237…” (PMID: 22584576), our elementary parsing technique would permit the detection of positions separated from the amino acid mention by linking terms, e.g. “serine *positions* 21”, as well as suites of figures, e.g. “21, 33, 230, and 237”. Extraction of the position of the modified site in the sequence is not mandatory, since this information is often found only in the full-text article. The putative modified protein was detected using the gene mention tagger AIIAGMT [17], which performed well in the gene mention task of the BioCreative II assessment [18]. AIIAGMT returns a list of potential candidate proteins (Figure 2) which is ranked according to a simple scoring schema (see Methods).

**Table 1:** PTM filtering tokens and information extraction assessment

**PTM**	**Filtering**	**Generic corpus**			**Positive corpora**	
	**token**	***# Filtered astracts***	***# Retrieved abstracts***	***Precision***	***# Abstracts***	***Recall***
Acetylation	“acet”	26,144	1,753	65%	97	89%
Amidation	“amid”	21,861	1,515	73%	61	95%
Disulfide bond	“disulf”	6,933	1,095	94%	514	75%
Glycosylation	“glyco”	31,379	2,746	73%	464	85%
Methylation	“methyl”	28,015	664	57%	47	87%
Phosphorylation	“phospho”	61,144	16,129	71%	906	93%
Sulfation	“sulf”	20,834	256	65%	40	92%

“Filtering token” is the term used to select the abstracts, “# filtered abstracts” is the number of abstracts which contain these terms, and “# retrieved abstracts” is the number of abstracts selected by the complete sentence extraction procedure. Precision was estimated based on manual analysis of 100 positive abstracts.

### Method assessment

For each PTM tested we estimated the precision on a generic corpus composed of all abstracts cited in UniProtKB/Swiss-Prot. This dataset was utilised both to refine the regular expressions used for PTM type and site identification and to define additional filtering rules - such as excluding DNA methylation. The precision was estimated by manual analysis of 100 retrieved abstracts which were not used for method refinement. The accuracy of protein identification by AIIAGMT was assessed independently of the PTM extraction, meaning that a PTM event was considered as correct although the actual protein target was not identified.

Table 1 provides a summary of the performance of the tool. Precision varies according to PTM type, reaching 94% for disulfide bonds (which have very precise extraction templates). The lowest precision was observed for protein methylation, due to the frequent occurrence of the “methyl” token in chemical compound names. For this particular PTM it is difficult to improve precision except by the time-consuming design of very fine-grained patterns and exclusion rules. The most frequent source of false positive sentences was due to the detection of terms wrongly interpreted as protein modification, such as “O-*acetylserine”* – a metabolite of cysteine synthesis – or “Sepharose 4B-*tyrosine*-*sulfa*nilamide” – an affinity chromatography chemical. Other errors were due to the detection of non-specific information, such as “…proteome-wide *lysine acetylation* has been documented in prokaryotes,…” (PMID: 22544907), although, in this case, the full-text article may contain PTM data for specific proteins. The detection of documents describing enzymes catalazing a specific PTM was also a frequent source of errors, e.g. “: Oligosaccharyltransferase (OST) transfers *glycan* to *asparagine* in the *N-glycosylation* sequon.” (PMID: 22559858). Finally, the lack of any syntactic parsing in our method also led to several identifiable errors. For instance, the title sentence: “Overexpression of Reactive *Cysteine*-Containing 2-Nitrobenzoate Nitroreductase (NbaA) and Its Mutants Alters the Sensitivity of Escherichia coli to Reactive Oxygen Species by Reprogramming a Regulatory Network of *Disulfide-Bonded* Proteins.” (PMID: 22564194), the system associates the cysteine mention to the disulfide bond bond although there is no dependency between these terms.

A strategy for improving the precision of identification would consist of further filtering the documents for information on the position of the PTM in the protein sequence. We noticed that the precision increases significantly when the position is also mandatory (data not shown), associated with a concomitant reduction in recall (since the abstract does not always contain this type of information). As the goal of the tool is to facilitate comprehensive annotation of PTMs (see “System applications”), and results are perused by human curators, we retained those abstracts which do not provide positional information for further checking.

The recall was estimated from the proportion of documents retrieved from a positive corpus that consisted of the set of abstracts cited in UniProtKB/Swiss-Prot as providing experimental evidence for the existence of the modification. Since UniProtKB/Swiss-Prot annotation is based on the full text of documents, it is likely that some of the information we are seeking is not available in the abstracts of this positive corpus. We therefore examined those abstracts that were not retrieved by the tool, in order to check if the abstract contained information on modified residues, or whether this information was restricted to the full text of the article. In the latter case, such “negative abstracts” were not scored as false negatives.

The evaluation of the gene/protein detection and ranking was performed by a manual check of 100 abstracts with PTM information. In 61, 23, and 4 documents the mention of the gene carrying the modification was ranked at the first, second and third position respectively. In only 12 abstracts did AIIAGMT fail to find the correct gene mention.

We also assessed our sentence extraction procedure on the GENIA project’s “PTM event corpus” (produced in a preparatory phase of the BioNLP Shared Task 2011, Epigenetics and Post-translational Modifications (EPI) task [19]) for three types of PTMs, acetylation, glycosylation and methylation (Table 2). The performance was evaluated based on the tools ability to find the type of PTM and its site (*event trigger* and *site* according to the BioNLP ST event representation), but not the protein target (*theme*) or the enzyme catalyzing the reaction (*cause*). For this reason, the assessment cannot be directly compared with the BioNLP event extraction results.

**Table 2:** Assessment on the GENIA and RLIMS-P corpora

	**Acetylation**	**Glycosylation**	**Methylation**	**Phosphorylation**
# events/documents	68	93	70	110
False negative	7	31	26	15
False positive	2	0	0	12
Recall	90%	66%	63%	86%
Precision	97%	100%	100%	89%

#events is the number of acetylation, glycosylation or methylation events with site information on in the GENIA corpus, #documents is the number of abstracts positive for phosphorylation information in the RLIMP-P corpus.

Phosphorylation information extraction was also evaluated on the RLIMS-P corpus [9] (Table 2, last column). This corpus provides a list of abstracts classified as positive or negative for protein phosphorylation information. Our tool has comparable precision to RLIMS-P, which relies on sophisticated information extraction techniques [10]. The lower recall can mainly be explained by our requirement for a site mention for a sentence to be selected. Actually in the RLIMS-P corpus, documents which contain only generic information on phosphorylation were classified as positive, such as the following title: “Phosphorylation, a factor controlling the synthesis of L-erythrodihydrobiopterin (BH2).” (PMID: 697844). We took advantage of the benchmarked gene normalization provided for each positive document of the RLIMS-P corpus to evaluate the gene/protein mention detection and ranking. Among the 95 positive documents our information extraction system classified correctly, the actual phosphorylated protein was mentioned at the first rank for 71 documents, at the second for 17, and at the third for 1. In only 6 cases was the correct protein not found because AIIAGMT did not correctly identify the protein name boundaries.

### System applications

The method we developed was designed to support the annotation of UniProtKB in two settings. The first application is the automated tracking of new publications describing protein modifications involving any of the seven target PTMs. The second application is the targeted retrieval of documents that provide potential supporting information for phosphorylation sites that were previously annotated in UniProtKB/Swiss-Prot based on information from high-throughput mass spectrometry-based proteomic studies.

#### A pipeline for screening PTM information

We set up a pipeline to automatically retrieve abstracts from PubMed that contain information on each of the seven target PTMs. In this pipeline, PubMed is queried with the keyword “protein”, using the Entrez Programming Utilities [20], and the resulting documents are processed by the PTM information extraction procedure. The selected abstracts (partitioned according to PTM type) are presented to curators in the form of an HTML document with relevant information such as the putative modifications, the putative modified amino acids(s) and their position(s), and gene mentions highlighted in the text (Figure 2). The ranked list of gene/protein mentions completes each abstract display. Since protein phosphorylation is frequently mentioned in abstracts even when no (new) sites are experimentally characterized in the associated publication, we retrieved only those abstracts that also mentioned a sequence position in the sentence. This reduced the number of extracted publications that provide information which is irrelevant to PTM annotation. Cumulative figures for monthly requests during a six month period are shown in Table 3. Although the number of retrieved phosphosites remains high, we found that many of these were already annotated in UniProtKB/Swiss-Prot. For the other types of PTMs, the number of retrieved abstracts remains manageable for human curators.

**Table 3:** Results of screening PubMed abstracts for PTM information

	
Retrieved abstracts	75,777
With PTM information	1,266 (863)
Acetylation	119 (56)
Amidation	96 (6)
Disulfide bridge	173 (42)
Glycosylation	108 (27)
Methylation	26 (6)
Phosphorylation	730 (730)
Sulfation	14 (2)

“Retrieved abstracts” are the result of querying PubMed with the keyword “protein”. The number of PTMs with positional information is shown in parentheses for each PTM type (site information was a prerequisite for retrieval of phosphorylation information).

#### Retrieval of supporting information for phosphosites annotated based on high-throughput proteomic studies

**Figure 3:**
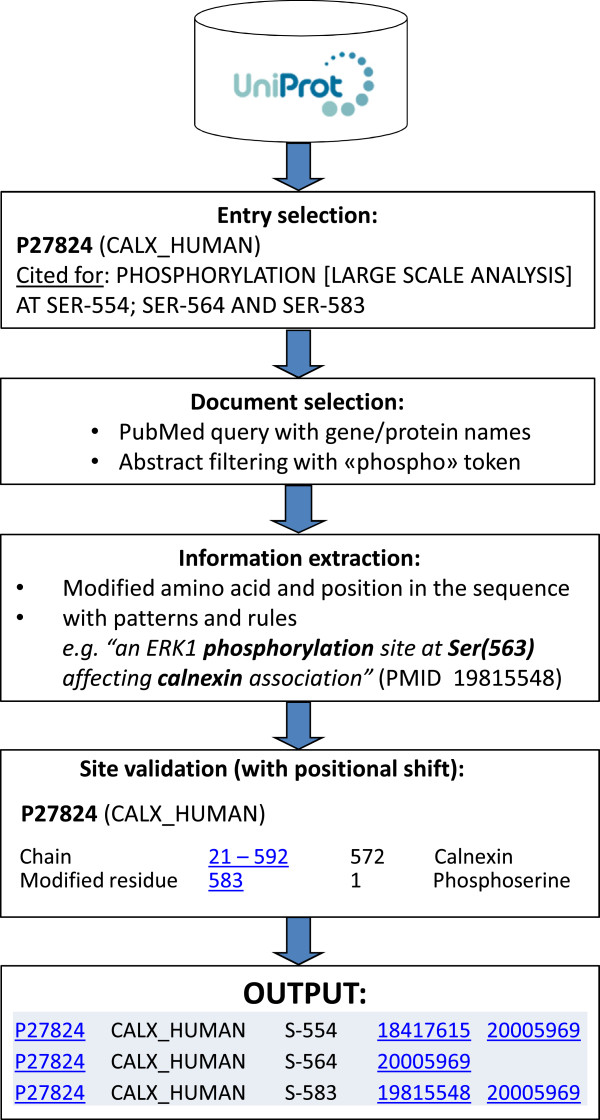
**Phosphosite information retrieval:** pipeline for the retrieval of documents that potentially provide supporting evidence for existing phosphosite annotations in UniProtKB/Swiss-Prot, where such annotations were made on the basis of information from high-throughput mass spectrometry-based proteomics experiments.

In UniProtKB/Swiss-Prot, over 14,000 entries contain experimentally determined phosphorylation sites whose positions were annotated based on information from high-throughput mass spectrometry-based proteomics experiments. These types of experiment often take the form of an exploratory survey, and do not include follow-up characterization to confirm the positions of the putative identified sites. We therefore implemented a procedure to scan the literature to find any new publications that may provide such confirmation (Figure 3). In brief, the procedure queries PubMed for each individual protein. The retrieved abstracts are then treated to extract information on phosphorylated sites. Each extracted site is then checked against the list of annotated sites in the corresponding UniProtKB/Swiss-Prot entry. The output of the pipeline is a tab-delimited file which displays the list of identified sites together with links to the corresponding UniProtKB/Swiss-Prot entries, and links to the abstracts from which the information was extracted.

When applied to the complete set of annotated phosphosites from high-throughput mass spectrometry-based proteomics experiments in UniProtKB/Swiss-Prot, our procedure retrieved abstracts matching 1,256 annotated phosphosites in 733 proteins. By manual analysis of the abstracts retrieved for 300 of these proteins, we estimated the precision of the procedure, calculated on unique sites, to be 66%. Most of the false positives were due to incorrect attribution of a modification of a conserved site found in a closely related homolog of the protein in question.

#### Sentence extraction from full-text articles

In order to assess the benefits of analyzing full-text articles, we performed a preliminary analysis using the open-access subset of UK PubMed Central (UKPMC) [21]. The papers were selected from the list of PubMed abstracts which were retained in the previous experiment following the first filtering step with the “phospho” token. We gathered over 11,000 articles from UKPMC that mention 4,350 proteins with phosphosites detected by high-throughput proteomics experiments*.* These articles were divided into sections and the sentence extraction procedure applied to each section. To reduce the number of false positive identifications we required that extracted sentences include a mention of the actual queried protein.

The information extraction system identified 228 articles with sentences which matched 167 phosphorylated sites annotated based on high-throughput proteomics experiments in 108 proteins. A precision level of 68% was calculated by manual analysis of the retrieved articles, with the majority of false positives again due to incorrect attribution of a modification to a homologous protein. As much as 90% of all matching sites were only described outside the abstract. Within the full text, around 38% of the extracted sentences were found in the “Results” sections, 29% in the “Methods” section (mainly associated with descriptions of antibodies on phosphorylated epitopes in proteins), 19% in the “Discussion” and 11% in the “Introduction”. The remaining 3% of sentences were found in undefined sections, mostly due to unusual sectioning. We also examined how a requirement that the queried gene/protein name and the phosphorylation site co-occurred in the same sentence would affect recall, by manually checking the extracted sentences from 50 articles. From the 239 sentences with validated site information, 53 were lost when this restriction was applied. However, for 33 of them, it did not result in a loss of information since the identified phosphosites were already found in other retained sentences of the same article.

This preliminary experiment demonstrates that full-text article analysis provides higher recall than simple abstract screening for PTM information extraction. It is difficult to draw general conclusions from this result though, as open-access articles available in XML represent only around 2% of the biomedical publications referred in PubMed. We therefore adapted the procedure to treat documents derived from the conversion in text format of the PDF version of articles. We downloaded from UKPMC a subset of 100 articles in PDF format, among the articles which show matching sentences in the previous experiment. From the 153 mentions of a phosphosite detected in these sentences, only 3 were not found in these converted articles, showing that our procedure is robust to errors due to PDF-text conversion.

We therefore carried out another experiment in the framework of a UniProtKB annotation task which consisted of trying to complete ambiguous citation content description where phosphorylation was mentioned without site specification. We screened 244 articles cited in 223 UniProtKB entries and were able to find 90 positions described in 48 articles. The precision was high, since there was no ambiguity concerning the modified protein; only 7 positions could not be added to the citation content description, mainly because of insufficient experimental evidence, a value judgment normally performed by a human curator. This last application is a clear demonstration of the complementarity of the text mining tools and the manual curation process.

## Discussion

The tool we present in this paper is designed to facilitate the annotation of information on protein post-translational modifications in UniProtKB. Our procedure offers a means to track publications describing new PTMs and can be used to link existing annotations to supporting evidence in the literature, which is vital for a number of applications including the cleaning of annotations derived from high throughput experiments and the development of evidence tags [22].

One of the main goals of text mining development is to increase the speed of database curation while maintaining the high accuracy of expert-curated annotations. Targeted surveys of curators suggest that appropriate text mining tools should be simple, user-friendly, and adaptable to the annotation needs of the particular database [2]. The role of the tool should be to facilitate the work of the curator; while it should retrieve relevant information at acceptable levels of precision and recall, it does not need to be perfect. Our system provides batch-processed results in a user-friendly format which facilitates visualization of the extracted information and access to additional resources. The process is simple and could be integrated in any curation workflow.

Our information extraction method integrates biological knowledge in the form of expert-curated patterns and domain-specific rules. Since it only relies on a specific vocabulary, the method can be easily extended to retrieve information on other type of PTMs. While such domain-specific approaches often achieve high precision at the expense of low recall, we purposely attempted to favor recall during the design of our tool, the output of which is provided to human curators. This was achieved by allowing non-specific pattern matches for PTM type detection and filtering of the resulting matches with additional rules restricting context and site specificity. In this context, we found that the identity of the actual modified position in the sequence significantly reduced the number of false positive identifications. A similar procedure proved equally useful in the development of a tool to retrieve new publications on protein mutations [23]. To detect phosphorylation events, RLIMS-P and MinePhos use similar pattern-matching approaches, but complement these with a prior NLP step consisting of part-of-speech tagging and phrase chunking. Although the performance of these tools were better than ours, a direct comparison was not feasible, as existing evaluations were performed on different datasets (for MinePhos), and the tool specifications were different (since RLIMS-P does not require the identification of the modified site). To extract PTM information, we deliberately chose to limit the use of natural language processing to very simple methods, such as propositional parsing, which does not significantly enhance the computational expense. It was a requirement of our system to perform large-scale document analysis in a reasonable time. Our tool has been used to treat nearly 1.5 million abstracts for validation of phosphosites detected by high throughput proteomics experiments. However, we did not measure of the impact of techniques, such as shallow or deep parsing, on the tool either at the level of extraction accuracy or expended computational time. Direct comparisons with the highest-performing methods for event extraction assessed in the BioNLP ST challenges are also not feasible, since the methods have different task specifications and site information was often not present in event annotation. Nevertheless our tool performs appropriately as a useful curator aid in a real-world annotation workflow.

Future developments will include consideration of methods to enhance the procedure for identification of the modified protein. This could be done normalizing the identified mention. Such normalization will allow the tool (1) to check if the detected residue at the detected position corresponds to the one in the protein sequence (2) to filter sites which are already annotated in the database. Gene normalization is a difficult problem, especially when only abstracts are analyzed since species information is often found in the core article. However, there has been progress in this task which was recently evaluated at the BioCreative III challenge [24], and we will test current programs for possible inclusion in our pipeline.

## Conclusions

This study presents a simple and robust procedure, based on domain-specific patterns and rules, to retrieve and extract from the biomedical literature information on protein post-translational modifications. Assessment of the method showed that it is competitive with other tools designed for the same purpose. The procedure was set up to treat both PubMed abstracts and full-text articles and to handle specific annotation tasks of the UniProt Knowldgebase. It demonstrates that text mining techniques can be efficiently applied in database curation.

## Methods

### Document pre-processing

Only those documents that contained at least one of the PTM-specific tokens (shown in Table 1) were selected for further processing. We added orthographic variants to some of the tokens, such as “(di)sulph” for “(di)sulf”. These documents were subsequently split into sentences using the MBT parser which was adapted for biomedical texts [25].

### Sentence and information extraction

The system extracts PTM information from retrieved documents in three distinct steps. First, the modification type detected in the pre-processing step using the PTM-specific token is sought in each document sentence using a set of specific regular expressions. These regular expressions were manually developed for each PTM using domain-specific knowledge resources such as the PSI-MOD ontology [26] and the controlled vocabulary of PTM descriptions of UniProtKB (http://www.uniprot.org/docs/ptmlist). They were deliberately designed to favor recall over precision, allowing some terms to be detected in contexts unrelated to protein modification, thereby increasing the rate of false-positive sentence retrieval. We analyzed these false positive sentences and used them to construct a list of specific terms that act as stop words, as well as a set of rules to detect their presence in appositives of the matching patterns. The second step involves identification of the modified amino acid and its position. For this we designed regular expressions for each amino acid at which a given modification can occur, as well as expressions designed to identify the N- or C-termini of the protein (for modifications which occur at the protein extremities such as acetylation, methylation and amidation), and some generic terms like “site” or “residue”. Finally, the modified site position in the protein sequence was detected by parsing the phrase downstream of the amino acid mention, in order to find specific coordinates that may be linked to it by elements such as prepositions, punctuations, and conjunctions. While identification of the modified amino acid is essential for a sentence to be retained, identification of the precise sequence position is not. The list of terms which were used for each of these steps is provided in a Additional file 1.

### Gene/protein mention detection

To identify modified protein(s) we analyzed the abstract using the gene mention tagger AIIAGMT [17]. The list of gene/protein names found by AIIAGMT was ranked according to a simple scoring scheme that takes into account the position of the gene/protein mention in the document relative to the information on PTMs. The score is incremented by +1 if the gene/protein is present in a retrieved sentence including information on PTMs, by +1 if it is present in the title, and by +1 if it is the most frequent gene/protein mention in the document. We did not attempt to normalize the gene names from a list of synonyms. This step is applied independently of the sentence extraction.

### Corpora

We used the following corpora for procedure development and testing purposes:

•A “generic corpus” that consisted of all PubMed abstracts from articles cited in UniProtKB/Swiss-Prot. This corpus consists of over 800,000 abstracts, and includes documents describing all aspects of protein function, not limited to PTMs, in a wide range of species with some bias to model organisms.

•A “positive corpus” that consisted of PubMed abstracts from articles that were cited in UniProtKB/Swiss-Prot as being articles that provide information on each type of PTM. These abstracts, gathered in the framework of the BioMinT project (available at http://biomint.isb-sib.ch), were used primarily to test system recall.

•A “PTM event corpus” of the GENIA project, which contains 157 PubMed abstracts with 405 PTM events, including acetylation, glycosylation, hydroxylation, and methylation (see [19] for a detailed description of the corpus). For assessment, we considered only events where the site was present.

•The RLIMS-P benchmarking dataset for phosphorylation that consists of 370 abstracts (110 positives, 260 negatives). Each positive abstract is linked to a UniProtKB entry (see [9] for description of the corpus).

### Retrieval of phosphosites detected by large scale experiments

Articles describing high-throughput mass spectrometry-based phosphoproteomics experiments can be easily identified within UniProtKB/Swiss-Prot as their citation scope (i.e. the text within the UniProtKB entry that provides the reason for which they are cited) is defined as “PHOSPHORYLATION [LARGE SCALE ANALYSIS]”. The citation scope also includes information on the nature of the modified amino acid(s) and the position(s), and this information was extracted from the XML version of the UniProtKB entries and used during validation. Other annotated sequence information of the UniProtKB record such as protein processing and alternative splicing events was also considered during position matching, as this may induce shifts in positional numbering within the protein sequence.

### PubMed query and abstract retrieval

A list of gene and protein names was extracted from each UniProtKB/Swiss-Prot entry containing phosphosites annotated based on information from high-throughput proteomic studies. This list was then used to query PubMed using the Entrez programming Utilities [20]. For non-human proteins the identity of the species was also included in the query. For human proteins, abstracts that contained mentions of other species were removed. For this purpose, species names and synonyms were extracted from the NEWT taxonomic database [27]. PubMed queries that returned no results were extended with other synonyms found in GPSDB, a thesaurus of gene/protein names and synonyms [28]. We limited the result set to a maximum of 20,000 documents, and removed gene and/or protein names that individually matched more documents than this (as these are potentially of low specificity). The matching abstracts were retrieved from a local implementation of MEDLINE using the EAGLi services (http://eagl.unige.ch/EAGLi), in order to reduce the search time.

### Full-text processing

We gathered 11,750 articles from the Open Access subset of UKPMC [21]. Documents having abstracts that included the “phospho” token were stored locally in XML format. Each article was then parsed in sections (“Introduction”, “Methods”, “Results”, “Discussion”, and “Unknown” for undefined sections) and the phosphorylation information extraction procedure was run on each section. For the sentence to be extracted, we required that the protein name that was used to generate the PubMed query which retrieved the article should also be mentioned. Names were defined by simple lookup of an exhaustive list of the gene and protein names and synonyms retrieved from GPSDB, together with automatically generated morphological variants.

When the PDF version of articles was used, we converted it to text using the Xpdf (http://www.foolabs.com/xpdf/). We then partitioned the document in sections using simple regular expressions and rules.

## Competing interests

The authors declare that they have no competing interests.

## Authors’ contributions

ALV developed and implemented the method. AB helped design the applications. JRM, JG and PR contributed to the processing of full-text articles. JG implemented the web interface. ALV and AB wrote the manuscript. LB and IX gave feedback to the manuscript. All authors have read and approved the manuscript.

## Supplementary Material

Additional file 1 **PTM vocabulary.** List of tokens used to create the regular expressions. Click here for file
