# Supplementary material for: Application of text-mining for updating protein post-translational modification annotation in UniProtKB
Source: BMC Bioinformatics. 2013 Mar 22;14:104. doi: 10.1186/1471-2105-14-104 (PMC3660268; doi:10.1186/1471-2105-14-104)
Supplement: Additional file 1 — PTM vocabulary. List of tokens used to create the regular expressions. [file 1471-2105-14-104-S1.pdf]

PTM vocabulary

The following tokens were used to construct regular expressions for PTM type and site detection, and to design exclusion rules. The expressions were implemented in a perl module with methods for each individual PTMs. The module is available on request from the authors. Examples of perl regular expressions are given.

1. **PTM specific terms**

Glycosylation

glyc[o|an]
s[i|y]al[i|y|o]
antennary
carbohydr
sugar
saccharid
Hex
hexos
Neu[5|N]Ac
NeuAc
NANA
N-acetyl-D-neuramic
N-acetyl-neuramic
N-acetylneuramic
N-acetyl-D-glucos
N-acetyl-glucos
N-acetylglucos
Glc
Gal
galactos
Xyl
xylos
Fuc
fucos
Man
manos
[N|O|C]-link
mucin-type

Perl regular expression:
$pattern =
qr/[Gg]lyc(?:o|an)|[Ss][iy]al[iyo]|[Aa]ntennary|[Cc]arbohydr|[Ss]ugar|[Ss]accharid|Hex|hexos|Neu(?:5|N)?Ac|NANA|N-acetyl(?:-|-D-)?(?:neuramic|glucos)|Glc|Gal\b|[Gg]alactos|Xyl|xylos|Fuc\b|[Ff]ucos|Man\b|[Mm]annos|[NOC]-link|[Mm]ucin-type/;

Methylation

methyl[*amino acid*], e.g. "methyllysine"

methyl-[*amino acid*]
methylated

Acetylation

acetyl
Ac-

Amidation

amid[e|at]
carboxyamid[e|at]
carboxy-amid[e|at]
[*1 or 3 letter amino acid codes*]-amide, e.g . “Gly-Gly-Leu-amide”
deamidat
carboxyNH2
carboxy-NH2
carboxyNH(2)
carboxy-NH(2)
[*1 or 3 letter amino acid codes*]-NH2, e.g. “RAILKNV-NH2”
[*1 or 3 letter amino acid codes*]-NH(2)

Perl regular expression:
```
$pattern = qr/(?:[Cc]arboxy-?|[A-Z]{3,}-?|[A-Z][a-z]{2}-
?|^)(?:(?:[Dd]e)?[Aa]mid(?:e|at)|NH\(?2\)*$)/;
```

Disulfide bridge

disulfide
disulphide
S-S

Phosphorylation

phosph[o|a]

Sulfation

sulf[a|o]
sulph[a|o]

PTM sites

- **Amino acids**
  full name, e.g. “lys(ine|yl)
  three letter code, e. g. “Lys”
  one letter code, e.g. “K”
  residue
  site

  Perl expressions (e.g. phosphorylation):

```
$pattern1 = qr/Arg|Asp|Cys|His|Ser|Tyr|Thr/;
$pattern2 = qr/\b(R|N|C|H|S|Y|T)[\-\(]?\d+?\b/
$pattern3 =
qr/([aA]rgin(?:ine|yl)|[aA]sparag(?:ine|yl)|[cC]yste(?:ine|yl)|[hH]istid(?:ine|yl)|[Ss]er(?:ine|yl)|[
Tt]hreon(?:ine|yl)|[Tt]yros(?:ine|yl)|[Rr]esidues?|[Ss]ites?)/;
```

- **N/C-terminus**
  Nterm
  N-term
  NH2-term
  NH(2)-term
  NH2 term
  NH(2) term
  amino-term
  amino term
  termin
  carboxy term
  COOH-term
  COOH term

Position detection

Number(s) sought after [*amino acid pattern*] + one or several linkage terms:
e.g. " two phosphorylated **serines** found at positions **235** and **239**"

Linkage tokens:
residue
position
and
at
in
is
are
was
were
located
found
[,|-|(|/]

2. **Exclusion terms (terms inducing sentence rejection)**

Glycosylation

glycolysis
[*glycosylation pattern*]ase
glycogen
[*glycosylation pattern*]phosph

phospho[*glycosylation pattern*]

Methylation

histone*
H[1|2|3|4]*
DNA
RNA
chromosom
chromatin]
inhibit
ADMA
symmetric
methylase
S-methyl
methy-d-aspartate
methyl adenosine
fluoromethyl
S-methyl
methylcoumar[in|yl]

Acetylation

histone*
H[1|2|3|4]*
acetylcholine
[*acetylation pattern*]-coA
[*acetylation pattern*]-coenzyme
[*acetylation pattern*]-gluc
[*acetylation pattern*]-glc
[*acetylation pattern*]-gal
[*acetylation pattern*]-neu
[*acetylation pattern*]-homoserine

Amidation

FMRFamide
FLRFamide
RFamide
ceramide
NH2-termin
NH(2)-termin

Phosphorylation

phosphatase
glucosphosphate
phosphoglucose
phosphoenol
phosphatydyl
phospholipid
[*amino acid pattern*] kinase

[*amino acid pattern*] phosphatase

Sulfation

inhibit
transport
[*sulfation pattern*]ase
dodecylsulfate
heparin-sulfate
chondroitin-sulfate
keratin-sulfate
dermatan-sulfate
ammoniumsulfate
sulfoxide
sulfolobus

Amino acid

S6
T cell
T lymphocyte

*sentences with information on histone methylation and acetylation are excluded since the sites are well-known.
